# Practical unidentifiability of a simple vector-borne disease model: Implications for parameter estimation and intervention assessment

**DOI:** 10.1016/j.epidem.2018.05.010

**Published:** 2018-05-26

**Authors:** Yu-Han Kao, Marisa C. Eisenberg

**Affiliations:** a Department of Epidemiology, University of Michigan, Ann Arbor, MI, United States; b Department of Mathematics, University of Michigan, Ann Arbor, MI, United States

**Keywords:** Dengue, Vector-borne disease, Mathematical modeling, Parameter estimation, Identifiability, Estimability

## Abstract

Mathematical modeling has an extensive history in vector-borne disease epidemiology, and is increasingly used for prediction, intervention design, and understanding mechanisms. Many studies rely on parameter estimation to link models and data, and to tailor predictions and counterfactuals to specific settings. However, few studies have formally evaluated whether vector-borne disease models can properly estimate the parameters of interest given the constraints of a particular dataset. Identifiability analysis allows us to examine whether model parameters can be estimated uniquely—a lack of consideration of such issues can result in misleading or incorrect parameter estimates and model predictions. Here, we evaluate both structural (theoretical) and practical identifiability of a commonly used compartmental model of mosquito-borne disease, using the 2010 dengue epidemic in Taiwan as a case study. We show that while the model is structurally identifiable, it is practically unidentifiable under a range of human and mosquito time series measurement scenarios. In particular, the transmission parameters form a practically identifiable combination and thus cannot be estimated separately, potentially leading to incorrect predictions of the effects of interventions. However, in spite of the unidentifiability of the individual parameters, the basic reproduction number was successfully estimated across the unidentifiable parameter ranges. These identifiability issues can be resolved by directly measuring several additional human and mosquito life-cycle parameters both experimentally and in the field. While we only consider the simplest case for the model, we show that a commonly used model of vector-borne disease is unidentifiable from human and mosquito incidence data, making it diﬃcult or impossible to estimate parameters or assess intervention strategies. This work illustrates the importance of examining identifiability when linking models with data to make predictions and inferences, and particularly highlights the importance of combining laboratory, field, and case data if we are to successfully estimate epidemiological and ecological parameters using models.

## 1. Introduction

Arboviral diseases are a global threat of increasing importance. Particularly for diseases propagated by *Aedes* mosquitoes, such as dengue, chikungunya, and Zika (Musso et al., 2015; Benelli and Mehlhorn, 2016), incidences have been increasing at alarming rates worldwide, with over 3.9 billion individuals believed to be at risk for dengue infection alone (Brady et al., 2012; Bhatt et al., 2013; World Health Organization, 2017). These increases are primarily attributed to the habitat expansion of *Aedes spp*. caused by changes in anthropogenic land use and human movement (Wilder-Smith and Gubler, 2008; Guzman et al., 2010; Gubler, 2011; Weaver, 2013; Wilder-Smith, 2012; Powell and Tabachnick, 2013). Given the ecology and life-cycle of *Aedes* mosquitoes, the transmission dynamics of these mosquito-borne diseases are heavily driven by complicated interactions between environmental factors (Hales et al., 2002, 2003; Scott and Morrison, 2003; Chen and Hsieh, 2012; Brady et al., 2014; Kraemer et al., 2015). These factors, combined with human behavior and transmission dynamics, make vector-borne diseases highly complex—presenting both challenges and opportunities for mathematical modeling (World Health Organization, 2011, 2012; Smith et al., 2012). Modeling has increasingly been viewed as a useful tool to quantify these complex transmission systems by integrating various data sources and specifying nonlinear mechanistic relationships and feedbacks. Numerous recent efforts at combating mosquito-borne diseases have directly incorporated the use of mathematical models, such as in planning for Zika and chikungunya response (Moulay et al., 2011, 2012a; Christofferson et al., 2016; Alex Perkins et al., 2016; Kucharski et al., 2016; Ferguson et al., 2016a), and evaluation of potential vaccine candidates (Chao et al., 2012; WHO-VMI, 2012; Aguiar et al., 2016; Ferguson et al., 2016b).

Indeed, mathematical modeling has a long history in vector-borne diseases, beginning with the original development of the Ross-Macdonald or so-called Susceptible-Infectious-Recovered (SIR) model to examine malaria (Kermack and McKendrick, 1927), and expanding to account for an enormous range of factors affecting both human and vector population dynamics (Andraud et al., 2012; Reiner et al., 2013). A wide range of modeling approaches, including ordinary and partial differential equations (ODE and PDE) (Enduri and Jolad, 2014; Aldila et al., 2013) as well as agent/individual-based models have also been applied to these questions (Li and Zou, 2009; Isidoro et al., 2011; Chao et al., 2012; Dommar et al., 2014; Manore et al., 2015). Common goals for many of these modeling efforts have been to make quantitative predictions of disease dynamics and to estimate the underlying mechanistic parameters (Chowell et al., 2007; Khan et al., 2014; Ferguson et al., 2016a; Perkins et al., 2016; Johnson et al., 2015).

To do so often requires using parameter estimation to connect these models with disease data, mainly using incidence or prevalence over time in humans. An important step in this process is examining parameter identifiability, the study of whether a set of parameters can be uniquely estimated and what parameter information may be gleaned from a given model and data set. Unfortunately, under many circumstances, the underlying model parameters are unidentifiable (also denoted non-identifiable), so that many different sets of parameter values produce the same model fit. The unidentifiability (non-identifiability) may be due to the model and measurement structure (i.e. structural non-identifiability) or the constraints of a specific dataset (i.e. practical unidentifiability). In either case, the data does not provide suﬃcient information for unique parameter estimation. Incorrect parameter estimates and ignorance of the uncertainty in prediction from an unidentifiable model can result in misleading epidemiological inferences, which could further lead to failures of public health interventions.

There are numerous transmission models in mosquito-borne diseases, which frequently use parameter estimation in fitting these models to data, and broader issues of parameter uncertainty and sensitivity have often been raised and explored (Johnson et al., 2015; Manore et al., 2014; Prosper et al., 2012; Reich et al., 2013; Mendes Luz et al., 2003; Laneri et al., 2010; Pandey et al., 2013; Shutt et al., 2017). However, relatively few efforts have been made to formally examine questions of parameter identifiability in these models (Mendes Luz et al., 2003; Laneri et al., 2010; Bhadra et al., 2011; Moulay et al., 2012b; Pandey et al., 2013; Reiner et al., 2014; Zhu et al., 2015; Tuncer et al., 2016; Shutt et al., 2017). Two studies that directly evaluated identifiability issues include: Denis-Vidal, Verdière, and colleagues assessed the structural (theoretical) identifiability of a chikungunya transmission model assuming all the states in human population and mosquito larva are observable (Moulay et al., 2012b; Zhu et al., 2015); Tuncer et al. (2016) examined both structural and practical identifiability of a within-to-between host model of Rift Valley fever, addressing how the multi-scale nature of such immuno-epidemiological problems affects model identifiability. Building on these results, we examine the identifiability of a simple compartmental model based on the Ross-Macdonald framework with various scenarios of measurement assumption (Newton and Reiter, 1992). This model is commonly used for both theoretical (Coutinho et al., 2005; Coutinhoa et al., 2006; Garba et al., 2008; Dumont et al., 2008) and applied epidemiological studies in a wide range of settings (Burattini et al., 2008; Yang and Ferreira, 2008; Chiroleu and Dumont, 2010; Pinho et al., 2010; Poletti et al., 2011; Sardar et al., 2016), and is often used in an expanded form where temperature or environmental dependence is explicitly included (Bartley et al., 2002; Yang et al., 2009a; Erickson et al., 2010; McLennan-Smith and Mercer, 2014). We consider the structural and practical identifiability of this model in the baseline case without explicit environmental drivers, using dengue incidence data in Kaohsiung, Taiwan as a case study. Additionally, the inclusion of mosquito population data has been considered helpful for parameter estimation in models involving mosquito life cycles (Reiner et al., 2013; Yang et al., 2009a; Morrison et al., 2011; Bowman et al., 2014). However, obtaining mosquito population data is diﬃcult in practice: it requires substantial time and resources which are often limited; spatial and behavioral variability in mosquito populations pose significant logistic challenges as well. Therefore, we also evaluate whether and to what degree that alternative mosquito data available in the field will reduce parameter uncertainty and improve model inference on mosquito control strategies. Finally, we present an example showing the consequences of ignoring unidentifiability in model-based intervention design.

Vector-borne disease modeling is often complex, and has been widely used in forecasting and the design of interventions (Ferguson et al., 2016a; WHO-VMI, 2012; Yakob and Clements, 2013; Patz et al., 1998; Focks and Barrera, 2006; Kearney et al., 2009). Through our simple model, we hope to draw attention to identifiability issues in vector-borne disease models and their implications in the application of models with more complexity.

## 2. Methods

**Fig. 1.**
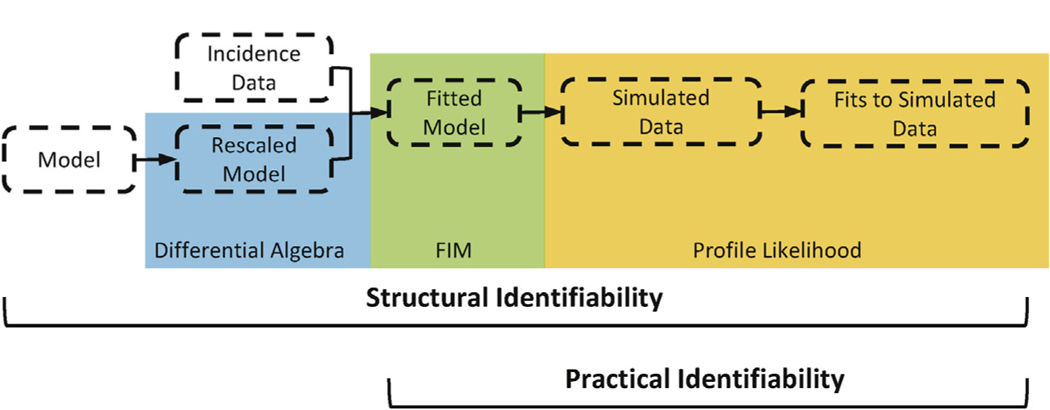
Summary of the parameter estimation and identifiability analysis process.

In the following sections, we will describe the model development, identifiability analysis, and parameter estimation processes. The flow chart in Fig. 1 summarizes the overall analytical process. The model and analyses were implemented in Python 2.7.10, with code available at https://github.com/epimath/dengue_model.

### 2.1. Model

**Fig. 2.**
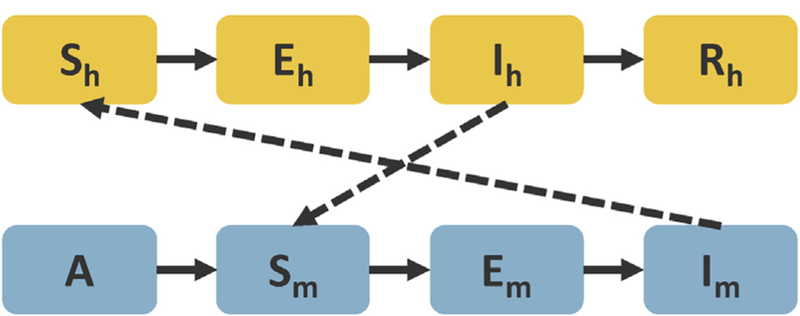
Diagram of the SEIR-based model. Subscript *h* indicates human, *m* indicates mosquitoes, and *S, E, I, R* represent susceptible, latent (exposed), infectious, and recovered humans or adult mosquitoes. A represents immature mosquitoes (larvae and pupae).

Our SEIR-based model is adapted from (Newton and Reiter, 1992; Yang et al., 2009a, 2009b), and shown in Fig. 2. We chose this model mainly because of its simplicity as well as its potential to be used for intervention design and epidemic prediction accounting for environment factors (Yang et al., 2009a, 2009b; Pinho et al., 2010; Poletti et al., 2011; Yakob and Clements, 2013; Erickson et al., 2010; McLennan-Smith and Mercer, 2014; Sardar et al., 2016). The model includes the disease transmission process between the human (*h*) and mosquito (*m*) populations. In addition, we specify an aquatic stage of mosquitoes combining larvae and pupae (*A*). These larvae/pupae then grow into adults (*S*_*m*_) and leave the aquatic environment. Since dengue virus is transmitted by the female mosquito, we only consider female mosquitoes in the model. The susceptible adult mosquitoes become infected and enter compartment *E*_*m*_ by having blood meals from infectious human beings carrying the dengue virus (*I*_*h*_). After the extrinsic incubation period (8–12 days) (World Health Organization, 2009; Chan and Johansson, 2012; Rudolph et al., 2014), the infected mosquitoes are capable of transmitting the virus and stay contagious during their lifetime (*I*_*m*_). Susceptible human individuals (*S*_*h*_) can be infected (*E*_*h*_) through bites from the mosquitoes, and then become infectious (*I*_*h*_) after a 4–10 day intrinsic incubation period (World Health Organization, 2009; Chan and Johansson, 2012; Rudolph et al., 2014). With proper treatment, individuals in the infectious stage can recover from dengue and are considered immune in the model. Note that multiple serotypes are not considered in the model, so potential interactions or antibody-dependent enhancement between serotypes are not included. We assume there is only mosquito-to-human and human-to-mosquito transmission in the model given the relatively low probability of other transmission pathways (World Health Organization, 2009).

#### 2.1.1. Model equations

In the model, we assume a constant human population (*N* = *S*_*h*_ + *E*_*h*_ + *I*_*h*_ + *R*_*h*_). We also consider all variables in units of individuals (i.e. humans, mosquitoes, and pupae/larvae).

(1) $$\frac{{\text{dS}}_{h}}{\text{dt}}=\mu \left(N-S_{h}\right)-\frac{\beta_{mh}S_{h}I_{m}}{N}\;\frac{{\text{dE}}_{h}}{\text{dt}}=\frac{\beta_{mh}S_{h}I_{m}}{N}-\alpha E_{h}-\mu E_{h}\;\frac{{\text{dI}}_{h}}{\text{dt}}=\alpha E_{h}-\eta I_{h}-\mu I_{h}\;\frac{{\text{dR}}_{h}}{\text{dt}}=\eta I_{h}-\mu R_{h}\;\frac{\text{dA}}{\text{dt}}=\text{ξ}\left(S_{m}+E_{m}+I_{m}\right)\left(1-\frac{A}{C}\right)-\pi A-\mu_{a}A\;\frac{{\text{dS}}_{m}}{\text{dt}}=\pi A-\frac{\beta_{hm}S_{m}I_{h}}{N}-\mu_{m}S_{m}\;\frac{{\text{dE}}_{m}}{\text{dt}}=\frac{\beta_{hm}s_{m}I_{h}}{N}-\text{γ}E_{m}-\mu_{m}E_{m}\;\frac{{\text{dI}}_{m}}{\text{dt}}=\gamma E_{m}-\mu_{m}I_{m}$$

It should be noted that *β*_*mh*_ and *β*_*hm*_ are transmission rates between host and vector populations, which are the products of average bites per mosquito and the probability of successful transmission per infected mosquito bite. *C* is the maximal carrying capacity of aquatic environment without the additional death term *μ*_*a*_ and maturation rate π. We also include a parameter to account for underreporting in human incidence and prevalence, so that the incidence in the model is measured as *y*_*h*_ *= κ*_*h*_*αE*_*h*_, where *κ*_*h*_ is the reporting fraction. Similarly, for counts and prevalence of mosquitoes, we assume that only a small fraction of the total mosquitoes are counted, assumed to be *κ*_*a*_ and *κ*_*m*_ for aquatic immature and mature mosquitoes, respectively. This yields the (simulated) observed immature mosquitoes to be *y*_*a*_ = *κ*_*a*_*A* and observed adult mosquitoes to be *y*_*m*_ = *κ*_*m*_(*S*_*m*_ + *E*_*m*_ + *I*_*m*_). Descriptions of the other parameters are given in Table 1.

#### 2.1.2. Rescaled model

Transmission models such as the one considered here can often be rescaled without changing the observed output. For example, in this model we could rescale the human variables to be larger (thereby also increasing the population size *N*), but reduce the reporting rate (*κ*_*h*_) and adjust the value of *β*_*mh*_ to yield the same apparent observed number of cases over time from the model. However, because each of these parameters (the reporting rate, transmission parameters, and size of the total population at risk) are all unknown parameters for our model, there is an inherent (structural) unidentifiability of these parameters, so that they cannot all be estimated simultaneously (i.e. for any population size, we can set *β*_*mh*_ and the reporting rate to yield the same observed number of cases). Similar issues can be found in the mosquito equations as well.

One way to correct these types of identifiability problems in the model is to rescale the model variables (e.g. *S*_*h*_*, E*_*h*_*, I*_*h*_*, S*_*m*_, etc.) by model parameters such as the total population size (in many cases this is equivalent to nondimensionalizing the system). In this case, we re-write the human model variables to be in terms of fraction of the population instead of numbers of individuals, e.g. letting the new variable for susceptible humans be: ${\tilde{S}}_{h}=S_{h}/N$ (and similarly for *E*_*h*_, *I*_*h*_, and *R*_*h*_). We also normalize the larvae *A* by their maximal carrying capacity C (letting $\tilde{A}=A/C$) and the remaining variables (*S*_*m*_, *E*_*m*_, and *I*_*m*_) by both *C* and π (i.e. letting ${\tilde{S}}_{m}=S_{m}/\left(C\pi \right)$*)* We note that this rescaling of the mosquito variables does not fully nondimensionalize them, but groups parameters into fewer terms (which is useful for identifiability purposes). Rewriting the equations and omitting the ∼’s yields:

(1) $$\frac{dS_{h}}{dt}=\mu \left(1-S_{h}\right)-\beta_{mh}^{*}S_{h}I_{m}\;\frac{{\text{dE}}_{h}}{\text{dt}}=\beta_{mh}^{*}S_{h}I_{m}-\alpha E_{h}-\mu E_{h}\;\frac{{\text{dI}}_{h}}{\text{dt}}=\alpha E_{h}-\eta I_{h}-\mu I_{h}\;\frac{{\text{dR}}_{h}}{\text{dt}}=\eta I_{h}-\mu R_{h}\;\frac{\text{dA}}{\text{dt}}={\text{ξ}}^{*}\left(S_{m}+E_{m}+I_{m}\right)\left(1-A\right)-\mu_{a}^{*}A\;\frac{{\text{dS}}_{m}}{\text{dt}}=A-\beta_{hm}S_{m}I_{h}-\mu_{m}S_{m}\;\frac{{\text{dE}}_{m}}{\text{dt}}=\beta_{hm}S_{m}I_{h}-\text{γ}E_{m}-\mu_{m}E_{m}\;\frac{{\text{dI}}_{m}}{\text{dt}}=\gamma E_{m}-\mu_{m}I_{m}$$

where $\beta_{mh}^{*}=\beta_{mh}C\pi /N,$ $\xi^{*}=\xi \pi ,$ and $\mu_{a}^{*}=\pi +\mu_{a}$. Similarly, the reporting rate parameters are now $\kappa_{h}^{*}=\kappa_{h}N,\kappa_{a}^{*}=\kappa_{a}C,$ and $\kappa_{m}^{*}=\kappa_{m}C\pi ,$ so that the observed human cases or mosquito counts are the same as in the original model. However, we note that this means that the reporting rate and population-at-risk can now only be estimated as a combined parameter (as is common for infectious disease models both vector borne and otherwise (Shutt et al., 2017; Eisenberg et al., 2013; Evans et al., 2005)). Rescaling allows us to reduce the number of parameters explicitly included in the model and correct some of the immediately apparent identifiability issues. We will show in Section 2.3 below that this also resolves the overall structural identifiability of the model

For the mosquito population of the rescaled model, we note that interventions involving removal of aquatic phase mosquitoes were conducted once the outbreak began. As a simplified way of representing these dynamics, we used the equilibrium assuming only logistic growth for the aquatic phase mosquitoes to calculate the initial conditions, and then simulated the model assuming an additional death/removal rate $\mu_{a}^{*}$ (although we note that technically, due to the rescaling, $\mu_{a}^{*}$ also includes the mosquito maturation rate). This yields initial conditions of 1 for *A* and 1/*μ*_*m*_ for *S*_*m*_. These initial conditions also gave a simple way to ensure the aquatic phase of the mosquitoes exhibited some timevarying behavior, since if *A* was started at the equilibrium using $\mu_{a}^{*}\neq 0,$ it would remain at equilibrium throughout the simulation (since there is no disease-related death in the mosquito population). We also tested the model when we assumed the initial conditions were at equilibrium with $\mu_{a}^{*}\neq 0$ a (yielding steady state values $A=\frac{\xi^{*}-\mu_{a}^{*}\mu_{m}}{\xi^{*}}$ and $S_{m}=\frac{\xi^{*}-\mu_{a}^{*}\mu_{m}}{\xi^{*}\mu_{m}})$, but this did not change our results (not shown).

#### 2.1.3. Basic reproduction number

The basic reproduction number (*ℛ*_0_) is the total number of secondary cases generated by introducing a single infected individual into a completely susceptible population(vanden Driessche and Watmough, 2002; Heﬀernan et al., 2005). Mathematically, *ℛ*_0_ is a threshold parameter controlling the stability of the disease-free equilibrium given by an entirely susceptible human and mosquito population. Using the next generation matrix (van den Driessche and Watmough, 2002), we construct *ℛ*_0_ as:

(3) $$R_{0}=\sqrt{\frac{S_{m}\alpha \beta_{hm}\beta_{mh}\gamma }{\left(\alpha +\mu \right)\left(\eta +\mu \right)\left(\gamma +\mu_{m}\right)\mu_{m}}}.$$

### 2.2. Parameter estimation

#### 2.2.1. Data

Weekly incidence of dengue cases since 1998 is available from the Taiwan National Infectious Disease Statistics System of Taiwan Centers for Disease Control (CDC) (Centers for Disease Control Taiwan, 2018). Confirmed dengue cases are reported from local hospitals and are released every week to the CDC online platform. In the study, we used 2010 dengue incidence data in Kaohsiung, the main city in southern Taiwan. Dengue outbreaks in Taiwan always start from and are often confined to the south because of the favorable environment for *Aedes spp*. Kaohsiung is usually the main epidemic area during outbreaks, and also has annual outbreaks regularly (Chang et al., 2012). The 2010 epidemic curve of dengue in Kaohsiung is very typical with one main peak. Since our model does not handle spatial heterogeneity and multiple strains, we chose to focus only on the 2010 data in Kaohsiung for these analyses.

#### 2.2.2. Parameter estimation

We neglect population birth/death dynamics in the model (*μ*=0) because the out break only lasts for 32 weeks. We also fix *α* and γ as 0.14 and 0.1 respectively based on previous studies (World Health Organization, 2009; Chan and Johansson, 2012; Rudolph et al., 2014), and let *η* be 0.1 since the infection usually lasts for about 10 days (World Health Organization, 2009). We estimated the remaining 6 parameters using weekly dengue incidence in Kaohsiung with least squares (i.e. maximum likelihood assuming normally distributed measurement errors). Nelder-Mead from NumPy in Python 2.7.10 was used for the estimation process.

#### 2.2.3. Simulated data

As discussed in identifiability analysis below, we also simulated noise-free data using the fitted model from previous step. These data were generated by simulating the given variables at either daily or weekly frequency. This allowed us to examine identifiability of the model in a case where the “true” parameters are known (so that errors in estimation can be assessed) and to consider a range of alternative measurement scenarios examining how adding diﬀerent types of mosquito count data might improve parameter identifiability. We synthesized the following four alternative simulated data sets corresponding to diﬀerent surveillance methods available in the field—dengue incidence, ovitrap/house index, BG-trap, and Gravid trap, respectively:

- Scenario 1: human incidence data only, given by *y*_*h*_=*κ*_*h*_*αE*_*h*_ (integrated to a weekly cumulative incidence)
- Scenario 2: human incidence data (*y*_*h*_) and daily aquatic (immature) mosquito counts, given by *y*_*a*_=κ_*a*_*A*
- Scenario 3: human incidence data (*y*_*h*_), aquatic mosquito counts (*y*_*a*_), and daily adult mosquito counts, given by *y*_*m*_=*κ*_*m*_(*S*_*m*_+*E*_*m*_+*I*_*m*_)
- Scenario 4: human incidence data (*y*_*h*_),aquatic mosquito counts (*y*_*a*_), and daily adult mosquito counts broken down by infection status, allowing us to break *y*_*m*_ into *y*_*ms*_ = *κ*_*m*_*S*_*m*_ and *y*_*mei*_=*κ*_*m*_(*E*_*m*_+*I*_*m*_).

We simulated these data in their most optimistic, best-case form—frequent measurements without noise. However, to examine how noise might aﬀect the parameter estimation, we also generated 300 simulated data sets for Scenario 1 with added measurement error based on the residuals from the parameter estimation with the Kaohsiung data (see Supporting Information).

#### 2.2.4. Estimation with simulated data

For parameter estimation using the simulated data, we fit the model with weighted least squares to account for the diﬀerent scales for mosquito and human data sets. The weights are the same for each point within each individual dataset (i.e. weighted by the average data value).

### 2.3. Identifiability analysis

We evaluated the structural and practical identifiability of the parameters, given the model and different possible data sets described above. We will give a brief overview of the identifiability definitions and methods used here. For a more complete review, please refer to (Cobelli and DiStefano, 1980; Audoly et al., 2001; Raue et al., 2009; Miao et al., 2011).

In general there are two types of identifiability: *structural identifiability* (sometimes just called *identifiability*), which examines the best-case scenario of perfectly measured, noise-free data, in order to reveal the inherent, theoretical identifiability derived from the model structure itself; and *practical identifiability* (sometimes called *estimability*), which examines how parameter identifiability fares when real-world data issues such as noise, sampling frequency, and bias are considered (Raue et al., 2009). When a model is unidentifiable, model parameters usually form *identifiable combinations*, which are combinations of parameters that are identifiable even though the individual parameters in the combinations are not.

#### 2.3.1. Structural identifiability analysis

We first examined structural identifiability using two approaches: differential algebra (Ollivier, 1990; Audoly et al., 2001; Pia Saccomani et al., 2001; Meshkat et al., 2012; Eisenberg et al., 2013) and the Fisher information matrix (Rothenberg, 1971; Cobelli and DiStefano, 1980; Cintrón-Arias et al., 2009; Eisenberg and Hayashi, 2014). A short overview of both methods, formal definitions, and examples are provided in the Supporting Information.

In brief, the differential algebra approach is an analytical method which examines whether it is possible, from the model equations and variables measured, to uniquely determine (estimate) the parameter values. The approach is based only on the model and data structure—it assumes perfect, noise-free data, without consideration of real-world issues of noise, bias, or sampling. This represents an idealized, best-case scenario; however many biological and epidemiological models are structurally unidentifiable, making this a useful first step in examining the parameter information available for a given model and data.

The differential algebra approach provides global results of model structural identifiability and closed forms of the relationships between parameters, but it is usually very computationally expensive. The Fisher information matrix (FIM) can be used as a numerical or analytical approximation to examine structural identifiability for a single point in parameter space (local results), for example, by using very finely sampled simulated data, as discussed in more detail in (Jacquez and Greif, 1985; Eisenberg and Hayashi, 2014). Given that the FIM is often used as a numerical rather than analytical method, there can be limited generalizability across the parameter space. However, it is significantly faster and less computationally intensive than the differential algebra approach.

Here, we test the four simulated data scenarios given above, using the differential algebra approach when possible (using both Mathematica code as well as the freely available packages COMBOS (Meshkat et al., 2014) and Daisy (Bellu et al., 2007)), and the FIM when the differential algebra approach was too computationally intensive to converge to a solution.

#### 2.3.2. Structural and practical identifiability using the profile likelihood

Another way to assess identifiability is the profile likelihood (Raue et al., 2009). Taking **p** = {*θ*_1_, …, *θ*_*p*_} as the parameters to be estimated, we fix a parameter (*θ*_*i*_) across a range of values, which is denoted as [*min*(*θ*_*i*_), *max*(*θ*_*i*_)], and fit the remaining parameters {*θ*_*j*_|*j* = 1, …, *p*, *j* ≠ *i*} using the likelihood function *ℒ* for each value of *θ*_*i*_ in [*min*(*θ*_*i*_), *max*(*θ*_*i*_)]. In our case, least squares is used to compute the best-fit values of *θ*_*i*_s, constituting the likelihood profile for the fixed parameter. A minimum in the profile likelihood indicates structural identifiability (at least locally). A parameter is structurally unidentifiable when its like-lihood profile is flat and is practically unidentifiable when the curva-ture of its likelihood profile is shallow (Eisenberg and Hayashi, 2014; Raue et al., 2009). However, the degree of shallowness for a profile is a question of degree, so there is often choice of where to set a threshold for practical unidentifiability. In order to decide whether the profile is “flat”, we constructed a 95% upper confidence bound for the profile likelihood given by: ${\hat{\sigma }}^{2}\chi_{0.95,p}^{2}$ where $\hat{\sigma }=\sqrt{\frac{\sum_{i=1}^{n}{\left(y_{i}-{\hat{y}}_{i}\right)}^{2}}{n-p}}$ with *n* denoting the number of observations, *p* the number of parameters to be estimated, and *y* and $\hat{y}$ the observations and model trajectory respectively (Raue et al., 2009). Using profile likelihood method, we examine the identifiability of the model with the four simulated data scenarios as well as the real dengue case data from 2010 in Kaohsiung, Taiwan.

## 3. Results

### 3.1. Model fitting and parameter estimation

**Fig. 3.**
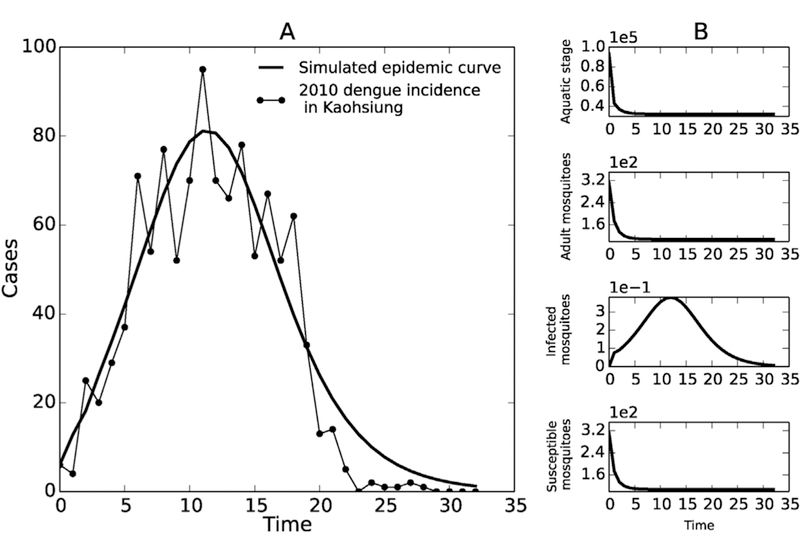
(A) model (dotted line) fitted to weekly incidence data (black circles) in Kaohsiung, Taiwan (2010); (B) simulated mosquito population data corresponding to Scenarios 2–4.

Using 2010 dengue incidence data in Kaohsiung, the fitted model was able to describe the general trend of the dengue epidemic. The left panel in Fig. 3 shows the dengue incidence data in 2010 and the fitted epidemic curve (*y*_*h*_). The model captures the overall epidemic size and the long tail at the end (though it overshoots for some of the tail). The fitted parameter values are given in Table 1. Some of the estimated parameters are on the edge of their biologically plausible ranges—for example *μ*_*m*_, the adult mosquito death rate, corresponds to a mosquito lifespan of approximately three days, which is within the reported range in the literature (Newton and Reiter, 1992; Burattini et al., 2008; Oki et al., 2011) (particularly with ongoing interventions), but short compared to most estimates. However, broadly, the estimated parameter values are diﬃcult to interpret, as the practical unidentifiability of the system (discussed below) means that we can shift the parameters significantly but still achieve the same fit.

As described in the methods, we also simulated both human and mosquito population data which is potentially collectible in the field. The simulated mosquito population data included *y*_*a*_ (aquatic stage), *y*_*m*_ (adult mosquitoes), and *y*_*ms*_ (susceptible mosquitoes) and *y*_*mei*_ (infected mosquitoes), shown in Fig. 3 (right panel). The fitted model and these simulated data were used for the following identifiability analyses.

### 3.2. Differential algebra and Fisher information matrix (FIM)

Using the differential algebra approach, we tested the best-case scenario including all the possible data sets from the field, i.e. Scenario 4: dengue incidence, aquatic mosquito counts, infected mosquitoes, and susceptible mosquitoes. With these four types of data together, we proved that the model is structurally identifiable. The detailed proof can be found in the Supporting Information section. However, we were not able to apply the differential algebra method to the remaining three scenarios, due to computational limitations. Therefore, we constructed the FIM to examine the structural identifiability of the model with all scenarios (Scenarios 1–4), using simulated, noise-free dengue incidence and mosquito counts. The FIMs for all the scenarios were full-rank (rank = 6, the number of parameters to be estimated), indicating that the model is locally structurally identifiable at the fitted values in Table 1.

### 3.3. Profile likelihood of estimated parameters

**Fig. 4.**
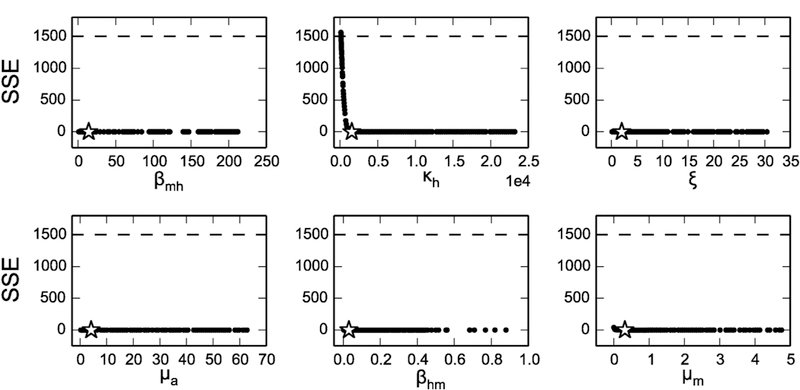
Profile likelihoods (black circles) assuming simulated, noise-free human incidence data (Scenario 1). Stars indicate the minimum sum of squared error (SSE) and dashed lines indicate the threshold for 95% confidence bounds. All six fitted model parameters are practically unidentifiable, with shallow minima which do not cross the confidence interval threshold within realistic biological ranges (zoomed in versions of the profiles showing the minima are given in the Supplementary Information).

**Fig. 5.**
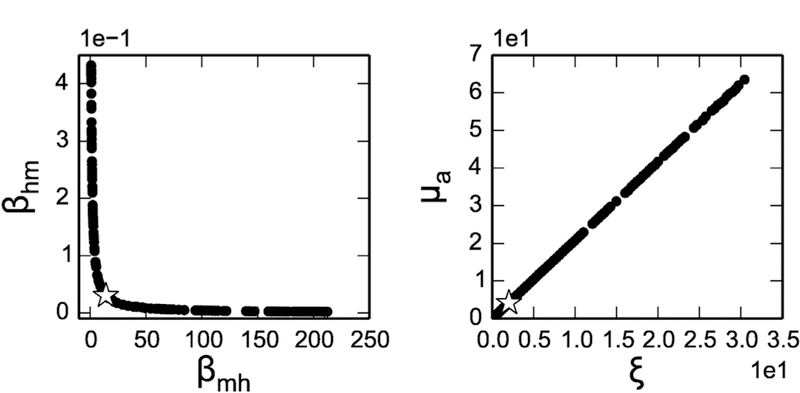
Parameter relationship scatter plots derived from the Scenario 1 profiles shown in Fig. 4, showing the relationships between *β*_*hm*_ and *β*_*mh*_ as *β*_*mh*_ is profiled and between ξ and *μ*_*a*_ as ξ is profiled. The two parameters in each pair compensate for one another, leading to the flat profile observed in Fig. 4.

The parameter profile likelihoods for both the dengue incidence data in 2010, Kaohsiung and the noise-free, simulated incidence data were very similar, with the Scenario 1 profiles shown in Fig. 4 and the Kaohsiung data in Supporting Information Figure S5. Taking *β*_*mh*_ in Fig. 4 as an example, the star represents the weighted sum of squared error (SSE) of the original fitted parameter values, and the dots are the SSE after adjusting the *β*_*mh*_ value and re-fitting the rest of the parameters. The dashed lines are the thresholds for the approximate 95% confidence bound of the profile likelihood. In principle, the profile likelihood curves of identifiable parameters should cross the thresholds on either side of the minimum (star), and the parameter values where they cross would be the confidence bounds. In this case, all the profiles are flat, meaning the fits are very similar regardless of the changing parameter values, and the confidence bounds are effectively infinite in one or both directions. This result would initially appear at odds with the structural identifiability of the model we showed earlier; however, upon zooming in the profiles, we can see there are minima in each profile (Supporting Information Figure S6). This suggests that although the model is structurally identifiable (consistent with the results from differential algebra and FIM approaches), it is not practically identifiable. To investigate the sources of this practical unidentifiability, we generated scatter plots of each pair of parameters, to evaluate whether any parameters are related to one another and form practically identifiable combinations. We were particularly interested in the pair *β*_*mh*_ and *β*_*hm*_—since they form a product in *ℛ*_0_, they could potentially compensate for one another and maintain the same overall magnitude of the epidemic. Indeed, these two parameters do appear to follow an approximate product relationship in their profiles, as illustrated in Fig. 5. In addition, there was a strong linear relationship between *ξ* and *μ*_*a*_, which are the parameters controlling the size of aquatic mosquito population. The remaining parameter relationships are shown in Supporting Information.

### 3.4. Profile likelihood with simulated mosquito data

**Fig. 6.**
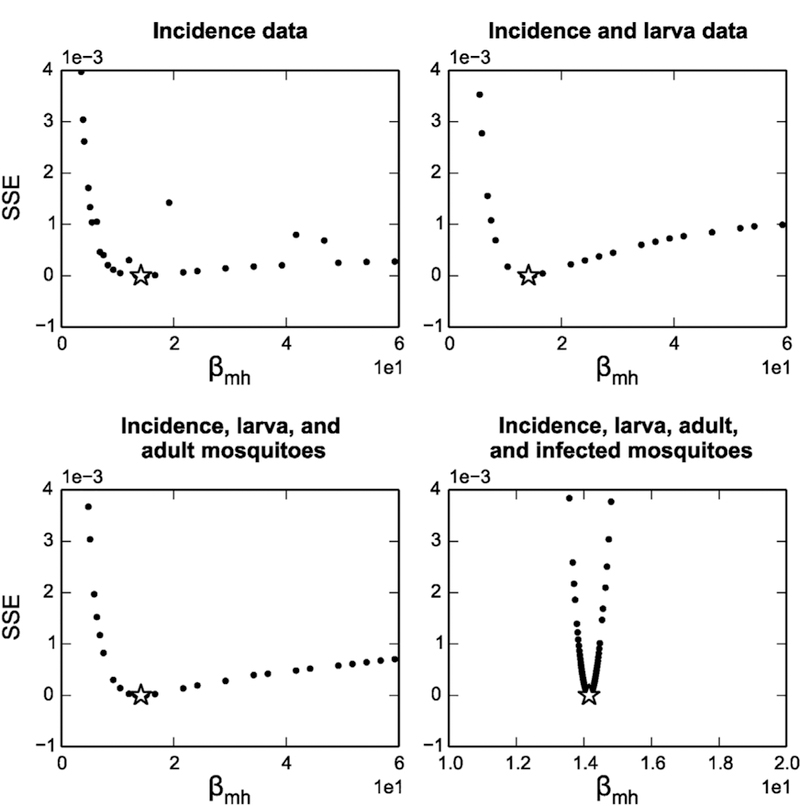
Profile likelihoods for *β*_*mh*_ with human incidence data only (Scenario 1), human incidence and larva count data (Scenario 2), human incidence, larva counts, and adult mosquito counts (Scenario 3), and data for human incidence, larva counts, adult mosquito counts, and infected adult mosquito counts (Scenario 4).

To evaluate whether including mosquito data collection could enhance model identifiability, we computed profile likelihood of the parameters using simulated mosquito population data sets (Scenarios 2, 3 and 4). A zoomed-in comparison between the *β*_*mh*_ profiles of Scenario 1 (only human incidence data), Scenario 2 (adding larva data), Scenario 3 (adding larva and adult mosquito data), and Scenario 4 (adding larva, adult mosquito and infected mosquito data) is shown in Fig. 6. The profiles were improved after adding mosquito information, as the curve slightly tilts up on the right-hand side and becomes higher on the left-hand side. However, the profiles including mosquito population data still do not exceed the 95% confidence threshold within a very wide range of *β*_*mh*_, implying that in practice there is not much obvious improvement on the profile likelihood after including mosquito surveillance data (Supporting Information Figure S4). We note that the small deviations from the profile curve are due to non-convergence of the estimation algorithm for some runs. The profiles for the remaining parameters are similar and are given in (Supporting Information Figure S4). The one exception to the overall trend of practical unidentifiability was that the reporting fraction parameter for the immature mosquitoes (*κ*_*a*_) was identifiable for all scenarios where mosquito data is measured (this parameter does not appear when only human data is used). The results using simulated noisy data in Scenario 1 (human data only) were very similar to those without noisy data, also showing flat profiles that did not reach the threshold for finite confidence bounds (see Supplementary Figure S3).

### 3.5. Profile likelihood with fixed parameters

**Fig. 7.**
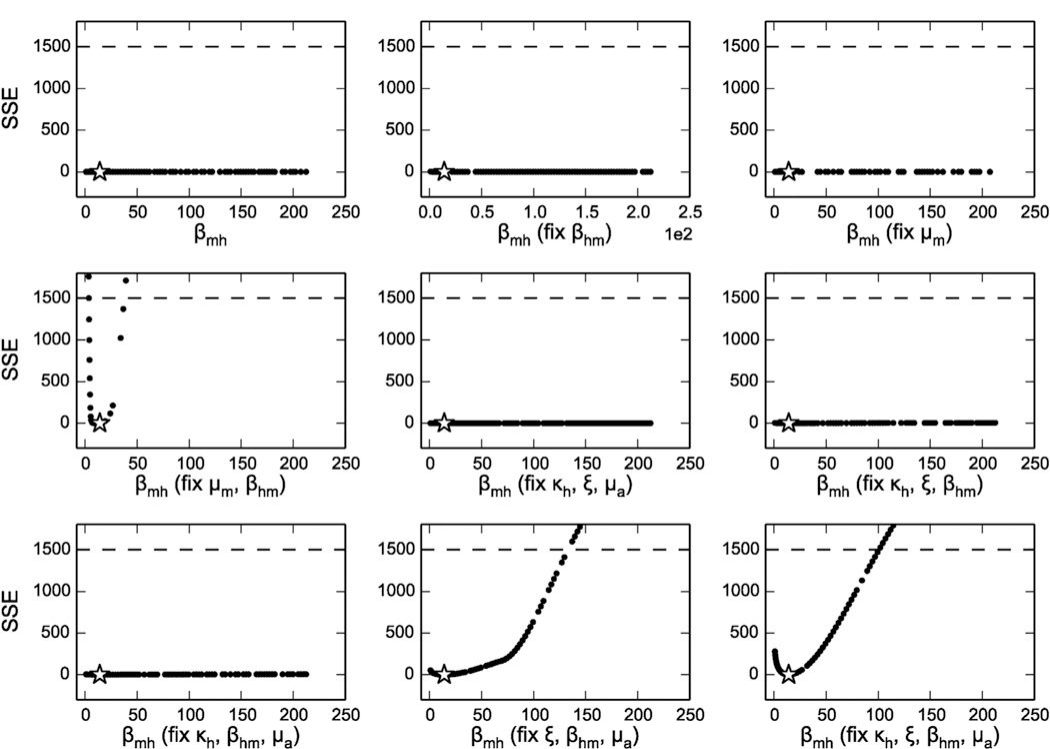
Profile likelihoods of *β*_*mh*_ when only subsets of *μ*_*a*_, *ξ*, *κ*, *μ*_*m*_ and *β*_*mh*_ are fitted. The fixed subset (in addition to *β*_*mh*_) is shown in parentheses on the *x*-axis.

Another way to resolve practical unidentifability is to decrease the number of parameters to be estimated, which can be done in the real world by having more information about specific parameters, such as using laboratory data to estimate the death rate for mosquito larvae. We examined this situation by fixing different sets of parameters to their originally fitted values (Table 1) and fitting the remaining parameters using synthesized dengue incidence data (Scenario 1). We demonstrate the results for the *β*_*mh*_ profile likelihood in Fig. 7. Given the relationship between *β*_*hm*_ and *β*_*mh*_, one might expect fixing *β*_*hm*_ could resolve *β*_*mh*_’s identifiability; nevertheless, the profiles indicate that fixing only one of the parameters appearing in ℛ_0_ (*β*_*hm*_ or *μ*_*m*_) is not suﬃcient to make *β*_*mh*_ identifiable. Fixing any of other combinations of the parameters not shown in ℛ_0_ does not improve *β*_*mh*_’s identifiability either. However, after fixing *β*_*hm*_ as well as either *μ*_*m*_ or the pair ξ and *μ*_*a*_, we obtained profile likelihoods with clear minima, crossing the confidence interval threshold, suggesting with a better idea or prior knowledge about these parameters, we can make *β*_*mh*_ identifiable. Unfortunately, as shown in Supporting Information, the whole model does not become identifiable until we fix at least four out of six parameters of interest.

The relatively small number of parameters in this model made it possible to near-exhaustively test a subsets of parameters to determine which ones yielded model identifiability. We started here with *β*_*mh*_ given the strong and apparent combination structure between the two transmission parameters (Fig. 5), and then tested fixing increasing subsets of parameters until we found subsets that resulted in finite confidence bounds. However, for larger models with more parameters, a more systematic approach would be needed, such as those presented in (Cintrón-Arias et al., 2009; Eisenberg and Hayashi, 2014; Balsa-Canto et al., 2010; Chis et al., 2011; Brun et al., 2002). A similar idea could also be incorporated in a Bayesian framework by adding suﬃciently strong priors to some of the unidentifiable parameters, which could allow successful estimation of the parameters. Indeed, this may be preferable in a real-world setting where parameter priors could be derived from the uncertainty in measuring the parameters through experimental/ecological studies. We note that due to the model unidentifiability, the estimation would thus rely heavily on the priors.

### 3.6. Basic reproduction number (ℛ_0_)

**Fig. 8.**
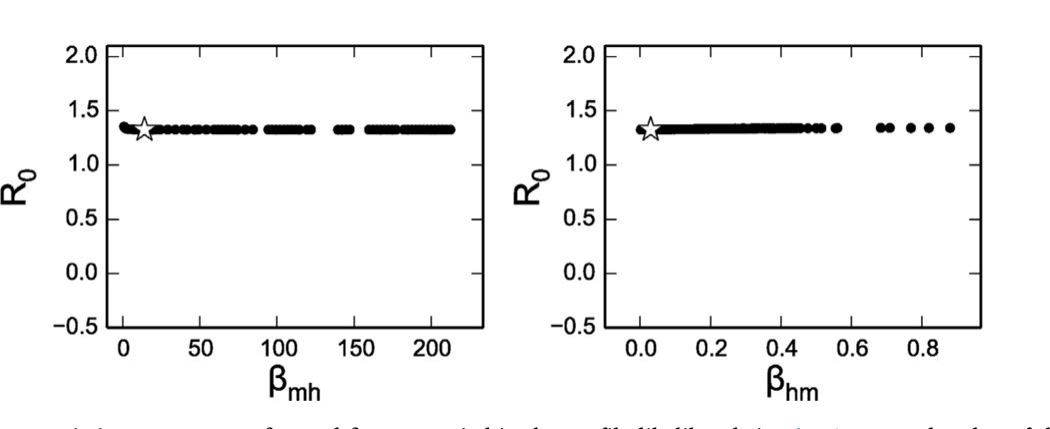
Values for *ℛ*_0_ as the two transmission parameters, *β*_*mh*_ and *β*_*hm*_ are varied in the profile likelihoods in Fig. 4. For each value of the profiled parameter, the plotted *ℛ*_0_ value uses to the best-fit values of the remaining parameters. *ℛ*_0_ remains relatively constant over the profiled parameter range, in spite of large changes in the parameter values.

Since *ℛ*_0_ is an important index for understanding disease transmission and predicting future epidemics, a key question is whether we can still estimate *ℛ*_0_ even when the model is practically unidentifiable. As an example exploration of this question, we calculate *ℛ*_0_ using Eq. (3), while profiling parameters *β*_*mh*_ and *β*_*hm*_, using Scenario 1 (human incidence data). Fig. 8 demonstrates that *ℛ*_0_ stays stable across the profile of *β*_*mh*_ and *β*_*hm*_ (the plots of the relationship between *ℛ*_0_ and other parameters are shown in Supporting Information Figure S7. The result indicates that we can often still obtain sensible *ℛ*_0_ estimates from the model with human incidence data, even though we cannot properly estimate the individual parameters.

### 3.7. Example intervention simulation

**Fig. 9.**
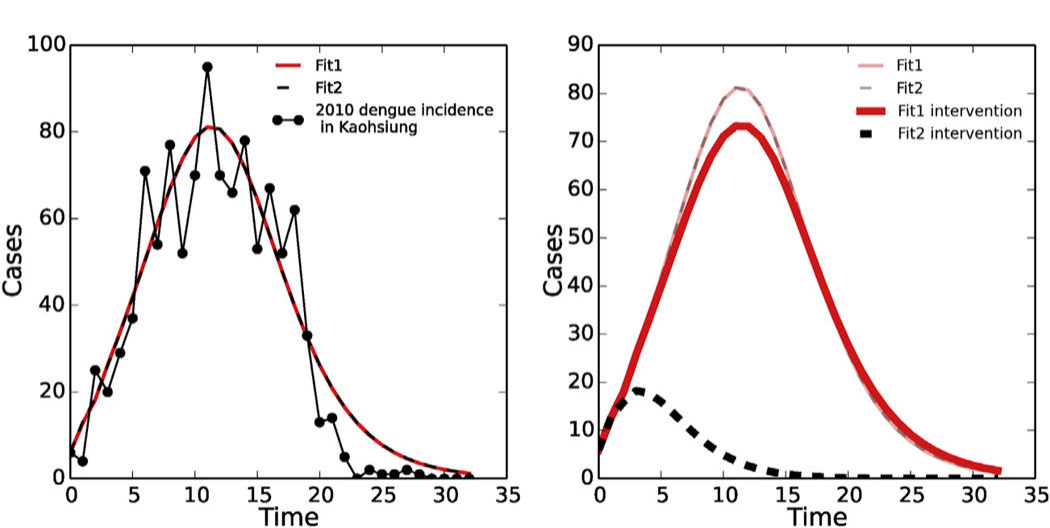
Illustration of the implications of model unidentifiability on intervention prediction. Left: two model simulations using different parameter values that give the same fit to data, based on the profiles in Fig. 4 (red solid line – original fitted parameter values from Table 1; black dashed line – [*β*_*mh*_ = 38.10, *κ*_*h*_ = 1625.42, *ξ* = 0.13, *μ*_*a*_ = 0.15, *β*_*hm*_ = 0.02, *μ*_*m*_ = 0.42]). Right: Simulated intervention results for both parameter sets, supposing that 10% of the aquatic (immature) mosquito population is removed at each time step.

We implement a very naive intervention in the model to demonstrate that ignoring unidentifiability can lead to misleading outcomes. We first pick two sets of parameters from the profile in Fig. 4 that generate very similar fits (shown in Fig. 9, left panel). We then remove 10% of the aquatic (immature) mosquito population each day to simulate the population control of mosquito larvae, which is a fairly common countermeasure against dengue. With the same implementation, the responses of the two parameter sets differ substantially: one epidemic curve only decreases minimally; however, the other simulation decreases significantly and dies out at an early stage of the out-break (Fig. 9, right panel).

## 4. Discussion

In this study, we explored both structural and practical identifiability of a commonly used SEIR-based model of vector-borne disease. We demonstrated that even when the model is structurally identifiable, it is likely to be diﬃcult or impossible to estimate both human and mosquito parameters from commonly available human incidence data in a single epidemic. In other words, although the likelihood surface of the model has a single optimum, it cannot practically be distinguished from a wide range or curve of neighboring points on the likelihood surface. Moreover, even in cases when human incidence data is combined with the types of mosquito data collected in the field, the practical identifiability of the parameters did not significantly improve. We then showed that more in-depth study of mosquito ecology and behaviors, which can give us direct information about individual parameters, was more eﬃcient in terms of improving model identifiability. Unfortunately, obtaining accurate measurements for any of these parameters individually can be very diﬃcult in practice, as they often vary depending on environmental and ecological factors such as temperature, weather events such as storms, and predation by other species (Kraemer et al., 2015; Wu et al., 2013; Morrison et al., 2008; Chang et al., 2011; Benelli et al., 2016) We would also need additional information on most of the parameters to make the model fully identifiable, which may not always be feasible. Nevertheless, it is still likely possible to measure the relative magnitude of some parameter subsets (e.g. identifiable combinations), providing constraints that can resolve the identifiability issues. For example, one could measure biological factors such as the relative infectivity from mosquitoes to humans and vice versa. This parameter, combined with knowledge of the human and mosquito population sizes (potentially even if only approximately known) could be used to constrain the ratio *β*_*mh*_/*β*_*hm*_ and resolve the identifiability of the two *β*’s.

The parameter analyses shown in Fig. 5 and Supplementary Figure S7 give additional guidance on which parameters may resolve the identifiability issues if measured, but more broadly, one could test fixing specific parameter sets using profile likelihoods for a wide range of models. The parameter sets tested might be based on what data is plausible to collect, or one could test parameter sets in a systematic way, e.g. using the identifiable combination structure and/or parameter parameter sensitivites (Cintrón-Arias et al., 2009; Eisenberg and Hayashi, 2014; Balsa-Canto et al., 2010; Chis et al., 2011; Brun et al., 2002). More generally, while we used a maximum likelihood approach here, examining what information was available from human and mosquito surveillance data alone, if parameters were measured using experimental/ecological studies, this information could be included in the model using a Bayesian approach wherein we use the uncertainty in the measured parameters to determine their priors. The fixed-parameter analyses given here can provide some sense of how a very strong prior for the measured parameters would constrain the parameter estimates and reduce uncertainty, but a Bayesian approach with real-world data may provide greater flexibility while still improving identifiability of the system.

In spite of these identifiability problems, the model generates very similar *ℛ*_0_ estimates across a range of profiled parameter values producing the same fit to the data (shown in Fig. 5 and Supplementary Figure S7). This means that estimation using the model may still be useful in characterizing the disease outbreak and spread, calculating vaccination coverage, and assessing the risk of vector-borne disease, even if the individual parameters cannot be determined. *ℛ*_0_ is an important measure that can be used to evaluate potential interventions in public health. For example, we can simulate a model that implements the intervention and compare the *ℛ*_0_ with and without the intervention to evaluate the potential effectiveness (e.g. by examining whether *ℛ*_0_ becomes less than one, or the magnitude of the reduction).

Nevertheless, we cannot solely depend on *ℛ*_0_ since it is possible to obtain very different predicted responses with the same intervention implementation, as shown in (Fig. 9). The two alternative parameter sets shown in Fig. 9 both fit the data equally well and have similar *ℛ*_0_ values (1.30 and 1.33), so that we cannot distinguish which of the predicted intervention responses is more likely. The intervention simulation used here is quite simple, but represents a commonly used control strategy. The example illustrates how a lack of consideration of parameter identifiability can potentially lead to significant errors in evaluating or comparing different intervention strategies.

This model is a simplified interpretation of vectorborne disease transmission, and only assumes one outbreak and a single viral strain. Despite this simple structure, we still cannot properly estimate the parameters from the model. Indeed, similar structural and practical identifiability issues have been noted even for simpler transmission models (Eisenberg et al., 2013; Evans et al., 2005; Cintrón-Arias et al., 2009; Tuncer and Le, 2018). Models with more complicated designs are often more likely to be unidentifiable, underscoring the importance of taking model identifiability into account before making any inferences from the model. Identifiability analysis allows us to understand what a model and data can really tell us, and can help with planning before we invest time and resources into a experimental or field study. Even if unidentifiability is inevitable, as long as we understand the behavior, uncertainty, and the limitations of the model, mathematical models can still be powerful tools to study disease transmission.

In the analyses presented here, we cover a set of basic and often overly optimistic scenarios, simulating data that is noise-free and frequently measured. Given these best-case scenarios, it is unlikely that real world data (which is likely less frequent and noisier) will improve the identifiability of the model. This is illustrated in Supplementary Figure S3 for human surveillance data, and is likely to be similar or worse for mosquito data, which is often noisy, diﬃcult to measure at the daily frequency simulated here, and would also include low prevalences of infection that are diﬃcult to detect. However, more comprehensive research is needed to investigate how issues such as different types of measurement and process noise, missing data, and data resolutions can further complicate parameter estimation. In many cases, these issues will likely further hinder the model parameter estimation and identifiability, but in some cases, more complex dynamics or process noise could potentially improve identifiability, making this a natural next direction for investigation. Nonetheless, this work shows that parameter estimation from incidence data alone is likely to be diﬃcult or impossible, highlighting the importance of integrating parameter information directly from experimental or field data. Given that such experimentally measured parameters usually vary as a function of environmental variables such as temperature and rainfall (Mordecai et al., 2013, 2017), future work to evaluate how model identifiability changes once this dependence is incorporated into the parameters would be a highly useful next step, particularly as previous studies have shown that uncertainty may vary over different temperature ranges (Johnson et al., 2015).

## Supplementary Material

1

## Figures and Tables

**Table 1:** Parameter estimates and values. Estimated parameters are marked in bold; confidence bounds and uncertainty for the fitted parameters are examined further below.

Parameter	Description (units)	Value	Source
μ	Human birth and death rate (day−^1^)	0	Newton and Reiter (1992)
***β_mh_***	Rescaled mosquito-to-human infection rate (day−^2^)	14.15	Fitted
α	Intrinsic incubation rate (day−^1^)	0.14	World Health Organization (2009), Chan and Johansson (2012), Rudolph et al. (2014)
η	Recovery rate (day−^1^)	0.2	World Health Organization (2009)
***ξ***	Rescaled oviposition-fertilization rate of larvae (day−^2^)	2.03	Fitted
***μ_a_***	Additionaldeathrateforaquatic(immature)mosquitoesduringtheepidemic,due to interventions and environmental changes (day−^1^)	4.18	Fitted
***β_hm_***	Human-to-mosquito infection rate (day−^1^)	0.03	Fitted
***μ_m_***	Mosquito death rate (day−^1^)	0.32	Fitted
γ	Extrinsic incubation rate (day−^1^)	0.1	World Health Organization (2009), Chan and Johansson (2012), Rudolph et al. (2014)
***κ_h_***	Fraction of cases reported multiplied by total human population at risk (number of individuals)	1546.74	Fitted
κ_a_	Maximum possible immature mosquito counts observed in traps: fraction of aquatic mosquitoes observed times total maximal carrying capacity of aquatic mosquitoes (used for simulated data only) (number of mosquitoes)	93,420	Wu et al. (2013)
κ_m_	Maximum possible observed growth rate of new adult mosquitoes: fraction of adults mosquitoes observed times the maximum maturation rate of mosquitoes (used for simulated data only) (number of mosquitoes/day)	98.71	Wu et al. (2013)
