# Supplementary material for: Practical unidentifiability of a simple vector-borne disease model: Implications for parameter estimation and intervention assessment
Source: Epidemics. Author manuscript; Available in PMC 2018 Dec 1. (PMC6264791; doi:10.1016/j.epidem.2018.05.010)
Supplement: 1 [file NIHMS1512189-supplement-1.pdf]

Supporting Information

Appendix S1. **Background on structural identifiability using differential algebra and the Fisher information matrix.**

Appendix S2. **Structural identifiability proof of Scenario 4 using differential algebra.**

S1 Appendix: Background on structural identifiabiltiy using differential algebra and the Fisher information matrix

Differential Algebra Approach

The differential algebra approach [1–4] is usually used to examine global structural identifiability; it can also be used to construct identifiable parameter combinations and reparameterize the model with these combinations, when the model is unidentifiable [5]. In general, the method aims at producing a set of monic polynomial equations from a model with only outputs (e.g. weekly dengue incidence and/or mosquito population data in our case), inputs (e.g. climate drivers; not included in our model), and their derivatives, plus parameters of interest (e.g. transmission rate and death rate) as coefficients in the equations. The set of equations is called the *input-output equations*, and we can obtain it simply through substitution and differentiation to eliminate unwanted variables in the model. The implication is that each output/measurement will have an input-output-equation, the coefficients of which are identifiable combinations for the model. From these combinations, we can compute whether the model parameters have a unique solution with the given output(s). Here, we give an example using a simple SIR model to illustrate this process (originally proved in [6]). Consider the model equations below:

$$\begin{aligned} \frac{dS}{dt} &= \mu - \beta SI - \mu S \\ \frac{dI}{dt} &= \beta SI - \gamma I - \mu I \\ \frac{dR}{dt} &= \gamma I - \mu R \\ y &= kI \end{aligned} \tag{S1}$$

where μ is birth and death rate, β is transmission rate, and γ is recovery rate. Prevalence (y) is the output of the model. We first rewrite the model with y ignoring the R compartment since it does not affect the model dynamics or the identifiability analysis results, which yields:

$$\frac{dS}{dt} = \mu - \beta S\frac{y}{k} - \mu S \tag{S2}$$

$$\dot{y} = \beta Sy - \gamma y - \mu y \tag{S3}$$

We then solve (S3) for S and its derivative ($\dot{S}$):

$$\begin{aligned} S &= \frac{\dot{y} + \gamma y + \mu y}{\beta y} \\ \dot{S} &= \frac{\ddot{y} + \gamma\dot{y} + \mu\dot{y}}{\beta y} - \frac{\dot{y}(\dot{y} + \gamma y + \mu y)}{\beta y^2} \end{aligned} \tag{S4}$$

Replacing (S2) with (S4) and clearing the denominators, we have:

$$-k\ddot{y}y + k\dot{y}^2 - \beta\dot{y}y^2 - k\mu\dot{y}y - (\gamma\beta + \mu\beta)y^3 - (k\gamma\mu + k\mu^2 - k\mu\beta)y^2 = 0 \tag{S5}$$

In order to obtain a monic polynomial equation, we divide (S5) by the coefficient of the highest ranking monomial of y, $-k$, yielding:

$$\ddot{y}y - \dot{y}^2 + \frac{\beta}{k}\dot{y}y^2 + \mu\dot{y}y + \frac{(\gamma\beta + \mu\beta)}{k}y^3 + (\gamma\mu + \mu^2 - \mu\beta)y^2 = 0 \tag{S6}$$

Eq. (S6) is the input-output equation, and there are four identifiable combinations (the coefficients of the input-output equation) for the SIR model. Assuming the four combinations equal to (a_1, a_2, a_3, a_4) respectively, solving for (β, k, γ, μ) in terms of (a_1, a_2, a_3, a_4) gives the unique solution for each parameter of interest, indicating that the SIR model is identifiable. More complete details about the differential algebraic method can be found in [2, 5]

Fisher Information Matrix

The Fisher information matrix F is a $p \times p$ matrix (where p is the number of parameters), which summarizes the amount of information contained in data about the parameters being estimated. If F is singular, the estimated parameters are unidentifiable. Furthermore, the rank of F represents the number of identifiable parameters or combinations. We calculate F using numerical approximation of the parameter sensitivities based on [7–9] as follows:
Let the model be

$$\begin{aligned} \dot{\mathbf{x}} &= f(\mathbf{x}, t, u, \mathbf{p}) \\ \mathbf{y} &= g(\mathbf{x}, t, \mathbf{p}) \end{aligned} \tag{S7}$$

where $\dot{\mathbf{x}}$ is a system of first order ODEs, with t representing time, and u the input functions (if any). $\mathbf{p} = \{\theta_1, \cdots, \theta_p\}$ is the set of parameters to be estimated, and the model outputs are assigned as $\mathbf{y}$. Then a simplified form of F for examining identifiability can be constructed as follows:

1. Construct the sensitivity matrix

   $$X = (s(t,\theta_1), \cdots, s(t,\theta_p))$$

   where $s(t, p_i) = \left[\frac{\partial y}{\partial p_i}(t_1), \cdots, \frac{\partial y}{\partial p_i}(t_n)\right]^T$

2. Compute the Fisher information matrix:

   $$F = X^T X$$

If F is full rank, the model is said to be locally structurally identifiable, while a rank deficient matrix indicates unidentifiability. In practice, we consider the parameters unidentifiable if the determinant of F is non-zero but small. Inverting F gives the Cramér–Rao bound covariance matrix corresponding to the lower bound on the variance of each parameter [8, 10]. This method can only give local and asymptotic estimates, which can be problematic with small amounts of data or models with an unusual likelihood surface.

S2 Appendix: Structural identifiability - Scenario 4 proof using differential algebra

Here we present the proof of structural identifiability for the model in Scenario 4. The calculations below are performed using Wolfram Mathematica 10.4.

Proposition S2.1. *The re-scaled model given in Eq. 2 is structurally identifiable for output equations describing dengue incidence (y_h), larvae/pupae (y_a), infected mosquitoes (y_{mei}), and susceptible mosquitoes (y_{ms}).*

Proof. Recall the measurement equations for Scenario 4 are:

$$\begin{aligned} y_h &= \kappa_h \alpha E_h(t) \\ y_a &= \kappa_a A \\ y_{mei} &= \kappa_m (E_m + I_m) \\ y_{ms} &= \kappa_m S_m \end{aligned}$$

We note that y_h would normally be integrated on a weekly basis to generate weekly incidence, but the differential algebra approach assumes perfect, noise-free, continuous measurements, we can simply differentiate the integrated data to yield the instantaneous incidence shown above, which (from a structural identifiability standpoint) contains equivalent information. From this, we can rewrite the model equations in terms of the measured variables, leaving only S_h, I_h, and I_m as unmeasured variables:

$$\begin{aligned} \frac{dS_h}{dt} &= \mu(1 - S_h) - \beta_{mh} S_h I_m \\ \frac{dy_h}{dt} &= \kappa_h \alpha \beta_{mh} S_h I_m - \alpha y_h - \mu y_h \\ \frac{dI_h}{dt} &= \frac{y_h}{\kappa_h} - \eta I_h - \mu I_h \\ \frac{dy_a}{dt} &= \xi \left(\frac{y_{ms} + y_{mei}}{\kappa_m} \right) (\kappa_a - y_a) - \mu_a^* y_a \\ \frac{dy_{ms}}{dt} &= \frac{\kappa_m}{\kappa_a} y_a - \beta_{hm} y_{ms} I_h - \mu_m y_{ms} \\ \frac{dy_{mei}}{dt} &= \beta_{hm} y_{ms} I_h - \mu_m y_{mei} \\ \frac{dI_m}{dt} &= \gamma \left(\frac{y_{mei}}{\kappa_m} - I_m \right) - \mu_m I_m \end{aligned}$$

The y_a equation is already in the form of an input-output equation (i.e. only in terms of the parameters, observed variables, and their derivatives). From the y_h, y_{mei}, and y_{ms} equations, we can solve for the S_h, I_h, and I_m variables, which we can then plug in to their respective equations to result in four equations only in terms of the measured variables, their derivatives, and the parameters, i.e. a set of input-output equations. One of these equations is the y_a equation above, however the other equations are quite large (the largest has over 4000 terms), so are omitted here for simplicity. From the coefficients of these equations, we can solve for each parameter individually, implying that the model is globally structurally identifiable, i.e. that it is possible to estimate all parameters of interest from all available human and mosquito surveillance data assuming no measurement errors. □

From this result, we can then conclude that the original, non-rescaled model (Eq. 1) was structurally unidentifiable—as the original parameters which were combined in rescaling can be changed (by compensating one parameter versus another), without altering the apparent outputs y_h, y_a, y_{mei}, and y_{ms}. The identifiable combinations in this case will simply be the parameters which were combined in the rescaling process, namely $\beta_{mh} C\pi/N$, $\xi\pi$, $\pi + \mu_a$, $\kappa_h N$, $\kappa_a C$, and $\kappa_m C\pi$. This

result can also be proven directly using the same method as in Proposition S2.1, and so we omit the proof here.

Corollary S2.1. *The non-rescaled model given in Eq. 1 is structurally unidentifiable for output equations describing dengue incidence (y_h), larvae/pupae (y_a), infected mosquitoes (y_{mei}), and susceptible mosquitoes (y_{ms}). In particular, the parameters $\beta_{mh}, C, \pi, N, \xi$, and μ_a are unidentifiable, forming combinations $\beta_{mh}C\pi/N$, $\xi\pi$, $\pi + \mu_a$, $\kappa_h N$, $\kappa_a C$, and $\kappa_m C\pi$. The remaining parameters are identifiable.*

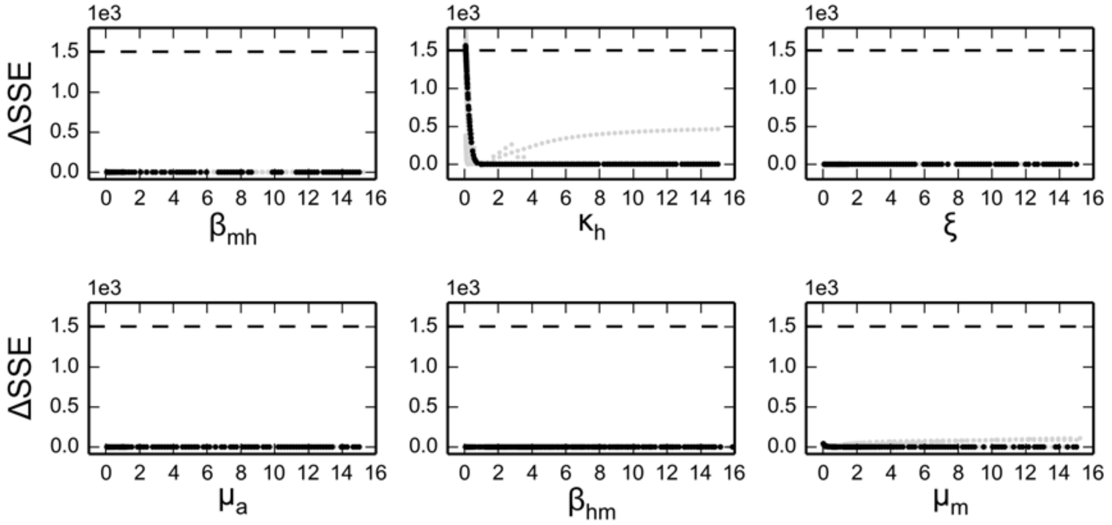

Figure S3. Profile likelihoods (grey circles) with 300 simulated data sets, assuming normally distributed measurement error with a standard deviation of 9.77 (derived from the residuals of the fit to the Kaohsiung data). Profile likelihoods from estimation using human case surveillance data from 2010 in Kaohsiung, Taiwan are shown as black circles. Dashed lines indicate the threshold for the 95% confidence intervals. The y-axes show the change in the sum of squared error (SSE) from the minimum SSE, and the x-axes show the fold change in each parameter. We note that two of the simulated data sets sets resulted in apparent deviation from the minimum SSE for the right-sides of the κ_h and μ_m profiles (still below the confidence interval threshold), likely due to the optimizer converging to a local minimum, or not fully converging. This can be seen as the optimizer sometimes reverted to the true (lower) best-fit minimum further along in the profile.

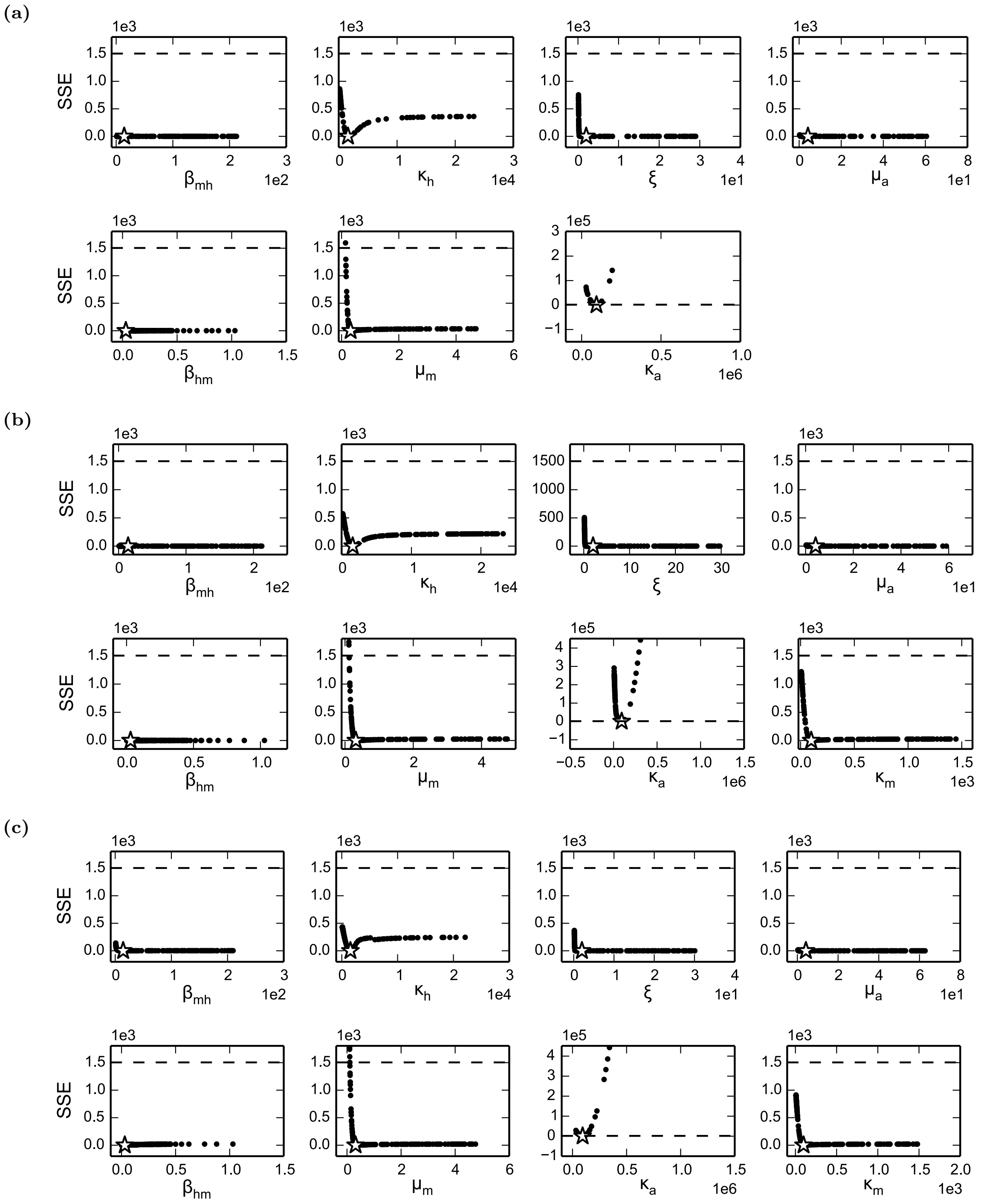

Figure S4. Profile likelihoods with simulated simulated human surveillance and mosquito data. Profile likelihoods (black circles) assuming simulated human case surveillance data as well as simulated data on: immature mosquito (larvae/pupae) counts (top panel), immature and adult mosquito counts (center panel), and immature and adult mosquito counts with adult mosquito infection status (bottom panel). Stars indicate the best-fit parameter value, and dashed lines indicate the threshold for the 95% confidence intervals.

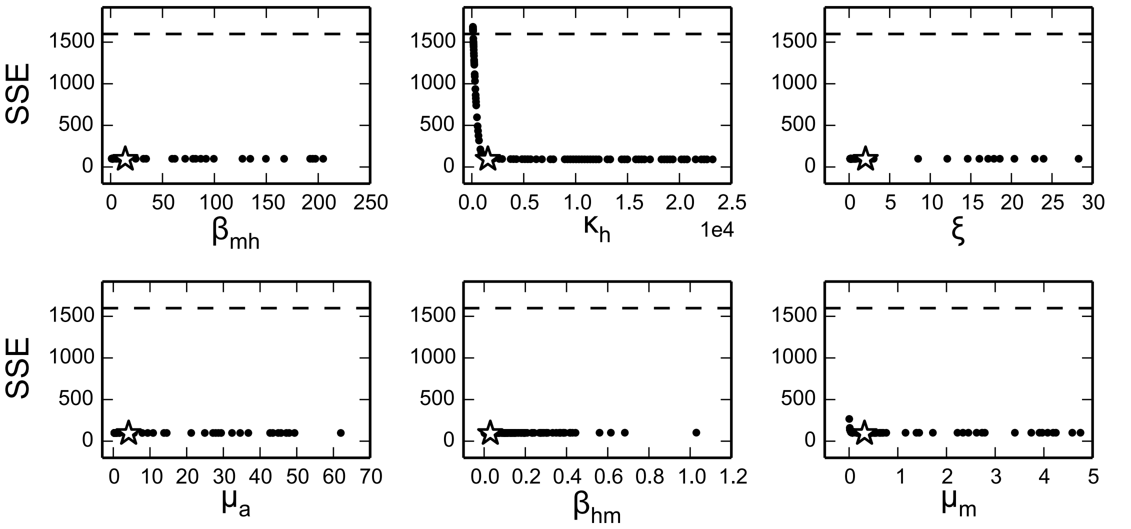

Figure S5. Profile likelihood with real human surveillance data. Profile likelihoods (black circles) with human case surveillance data from 2010 in Kaohsiung, Taiwan. Stars indicate the best-fit parameter value, and dashed lines indicate the threshold for the 95% confidence intervals.

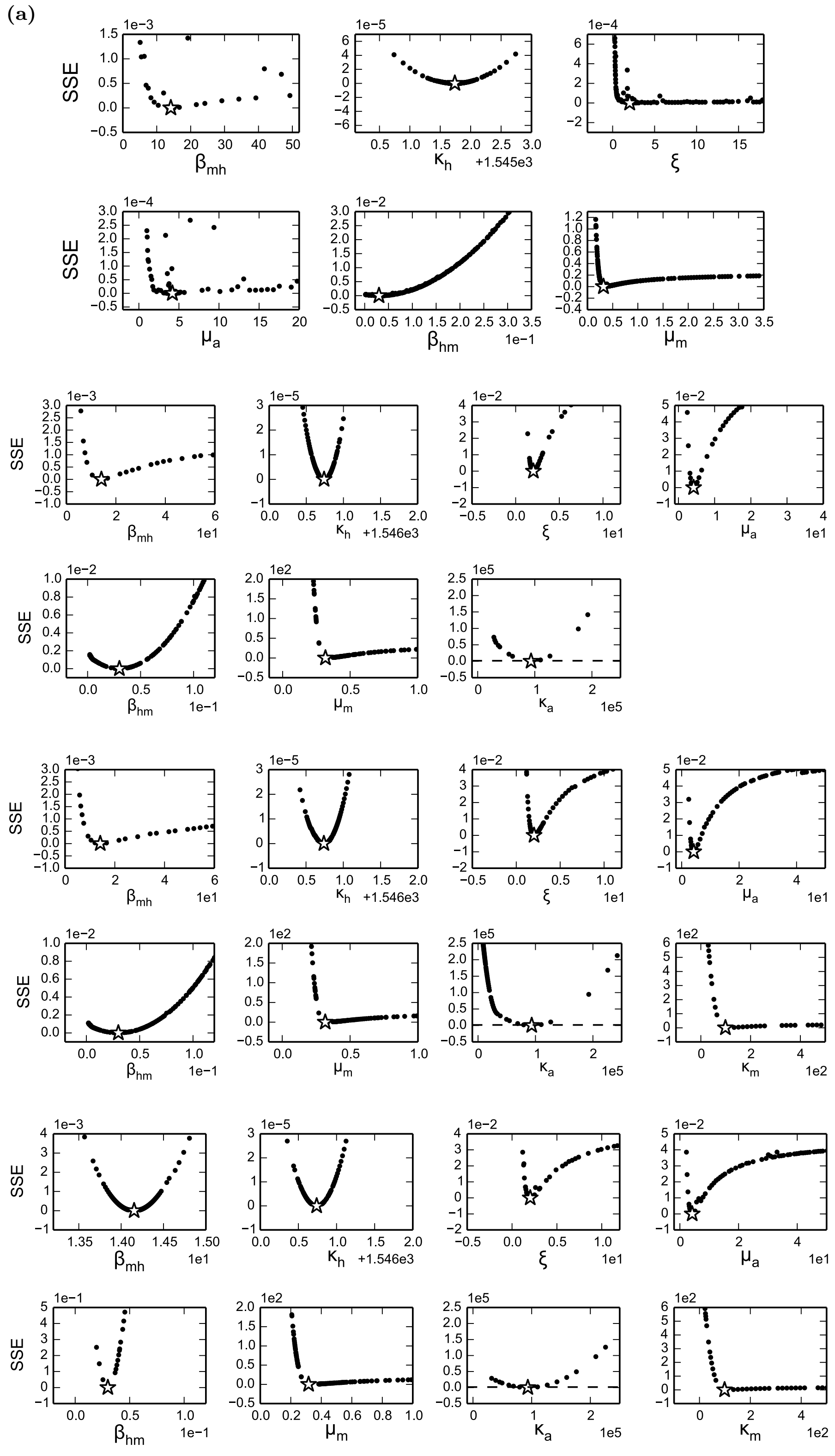

Figure S6. Zoomed-in profile likelihoods. Zoomed-in views of the profile likelihoods in Figure 4 and Supporting Information Fig. S4, illustrating the minima surrounding the best fit values (best-fit parameter values given as stars): (a) assuming simulated human case surveillance data, (b) adding mosquito (larvae/pupae) counts, (c) adding immature and adult mosquito counts, and (d) adding immature and adult mosquito counts with adult mosquito infection status.

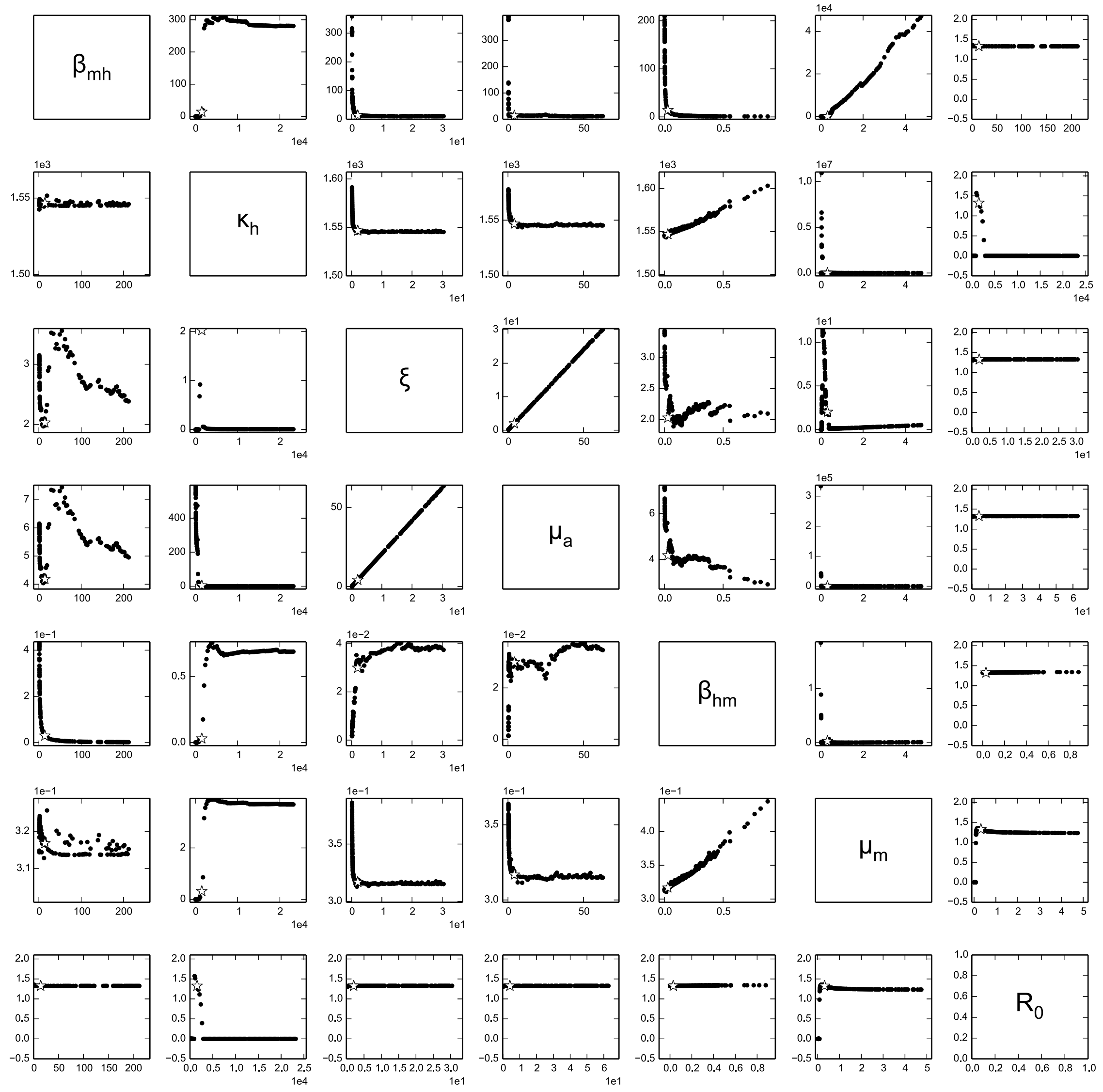

Figure S7. Parameter relationships for human surveillance data profile likelihoods. Compensatory relationships between parameters as each parameter is profiled, assuming human surveillance data. Some parameters show pronounced and consistent relationships with one another, indicating the possibility of a practically identifiable combination, while other parameters appear to have no relationship or noisy/inconsistent relationships depending on which parameter is profiled (possibly indicating more complex combination structures between multiple parameters rather than a single pair).

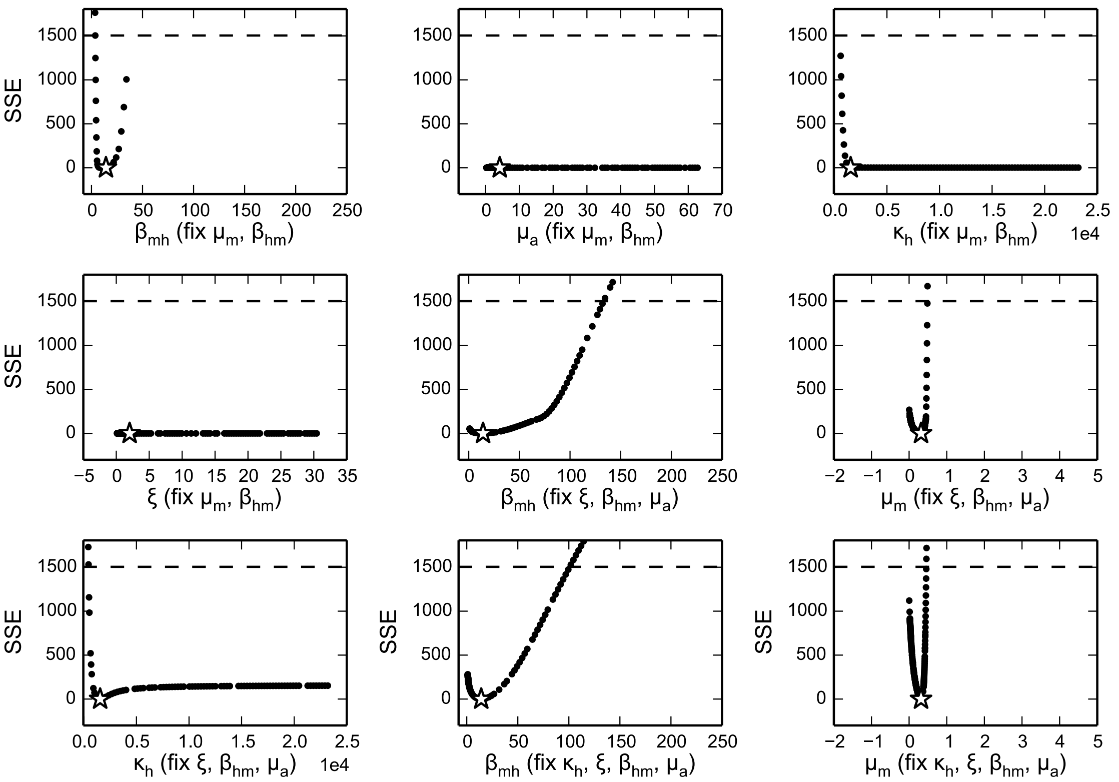

Figure S8. Profile likelihood when fitting subsets of parameters. Profile likelihood (black circles) when fixing 1) μ_m and β_{hm}; 2) β_{hm}, μ_a, and ξ; 3) β_{hm}, κ_h μ_a, and ξ. Stars indicate the best-fit parameter value, and dashed lines indicate the threshold for the 95% confidence intervals.

References

1. Ollivier F. Le probleme de l'identifiabilite structurelle globale : approche theorique, methodes effectives et bornes de complexite. École Polytechnique; 1990.

2. Audoly S, Bellu G, D'Angiò L, Saccomani MP, Cobelli C. Global identifiability of nonlinear models of biological systems. IEEE Trans Biomed Eng. 2001;48(1):55–65. doi:10.1109/10.900248.

3. Pia Saccomani M, Audoly S, Bellu G, D'Angio L. A new differential algebra algorithm to test identifiability of nonlinear systems with given initial conditions. In: Proc. 40th IEEE Conf. Decis. Control (Cat. No.01CH37228). IEEE; 2001. p. 3108–3113. Available from: http://ieeexplore.ieee.org/lpdocs/epic03/wrapper.htm?arnumber=980295.

4. Meshkat N, Anderson C, DiStefano III JJ. Alternative to Ritt's pseudodivision for finding the input-output equations of multi-output models. Math Biosci. 2012;239(1):117–123. doi:10.1016/j.mbs.2012.04.008.

5. Miao H, Xia X, Perelson AS, Wu H. On identifiability of nonlinear ODE models and applications in viral dynamics. SIAM Rev. 2011;53(1):3–39. doi:10.1137/090757009.

6. Evans ND, White LJ, Chapman MJ, Godfrey KR, Chappell MJ. The structural identifiability of the susceptible infected recovered model with seasonal forcing. Math Biosci. 2005;194(2):175–197. doi:10.1016/j.mbs.2004.10.011.

7. Rothenberg TJ. Identification in Parametric Models. Econom J Econom Soc. 1971;39(3):577–591.

8. Cintrón-Arias A, Banks HT, Capaldi A, Lloyd AL. A sensitivity matrix based methodology for inverse problem formulation. J Inverse Ill-posed Probl. 2009;17(6):545–564. doi:10.1515/JIIP.2009.034.

9. Eisenberg MC, Hayashi MAL. Determining identifiable parameter combinations using subset profiling. Math Biosci. 2014;256:116–126. doi:10.1016/j.mbs.2014.08.008.

10. Cobelli C, DiStefano JJ. Parameter and structural identifiability concepts and ambiguities: a critical review and analysis. Am J Physiol. 1980;239(1):R7–R24.
